# Precolumn Derivatization High-Performance Liquid Chromatography for Determination of Perfluorocarboxylic Acids in Catalytic Degradation Solutions

**DOI:** 10.1155/2022/3482759

**Published:** 2022-05-19

**Authors:** Liping Yang, Binbin Sun, Haochen Cui, Lingyan Zhu, Guoqiang Shan

**Affiliations:** Key Laboratory of Pollution Processes and Environmental Criteria (Ministry of Education), Tianjin Key Laboratory of Environmental Remediation and Pollution Control, College of Environmental Science and Engineering, Nankai University, Tianjin 300350, China

## Abstract

Perfluoroalkyl carboxylic acids (PFCAs), a series of ubiquitous contaminants in the global environment, attracted much attention due to their potential for high bioaccumulation and toxicity to various organisms. There are a lot of measurement requests in currently increasing degradation studies of PFCAs, which usually rely on expensive liquid chromatography-mass spectrometry (LC-MS). The degradation solutions containing high-concentration PFCAs can easily cause the pipeline pollution of the LC/MS instrument, which is usually used for trace analysis of environmental samples. In this study, a simple and reliable precolumn derivatization LC method coupled with an ultraviolet detector (UV) was developed for the determination of the main PFCAs (C_4-9_) of environmental concern. These PFCAs in degradation solutions were crosslinked to UV-responsive 3, 4-diphenylamine (DCA) by a carbodiimidization method, followed by a simple solid-phase extraction (SPE) cleanup, and quantitatively measured using a conventional LC-UV instrument. Compared to previously reported precolumn derivatization methods, this new derivatization approach has the advantages such as mild reaction conditions, easy operation, enhanced stability of derivatives, and low cost. The instrumental limits of detection (ILDs) for the targeted PFCAs in organic and aqueous mediums were 0.2–0.5 and 0.6–1.5 mg/L, respectively. The method has been successfully applied to the determination of PFCAs in catalytic degradation solutions and recommended for use in other assays involving relatively high-concentration PFCAs.

## 1. Introduction

Perfluoroalkyl carboxylic acids are a series of artificial perfluorinated fatty acids usually with 4–14 carbon chains [1]. Because of their superior surface properties, as well as high chemical or thermal stability, they have been widely used in industrial processing and for the production of a variety of commercial products since the 1950s [1]. Due to such large-scale applications and reaction inertness in the environment, PFCAs have been inevitably released into the natural environment and have become a class of ubiquitous contaminants worldwide [2]. Among these PFCAs, perfluorooctanoic acid (PFOA), historically the most productive and most prominent pollutant species, was included in Appendix A by the Stockholm Convention in 2019, since numerous studies unveiled that it shares the characteristics of the initial “dirty dozen” persistent organic pollutants (POPs), such as persistence, bioaccumulation, and toxicity [3]. With the ban or phasing-out of PFOA, many studies have suggested that short-carbon chain PFCAs can serve as PFOA substitutes which would lead to their more prominent application and likely contribution to environmental contamination [4]. Therefore, field monitoring and laboratory investigations of PFOA and its short-chain homologs should be prioritized to explore their environmental behaviors, toxicology, and degradation processing [5].

In the aforementioned works, a variety of analytical techniques were performed for determination of PFCAs in the samples both from field surveys and laboratory experiments [6, 7]. Low-volatility PFCAs can be dissolved and dissociated to carboxylate anions due to their relatively low p*K*_a_ values [8], which is ideal for liquid chromatography (LC) separation. However, due to a lack of strong ultraviolet (UV) chromophores in their structure, PFCAs are not be easily identified by LC-UV [7]. Although there were a few reports that directly adopted LC-UV for detection of PFCAs *via* a UV signal of nonspecific wavelengths under 210 nm [6, 8, 9], the LC determination of PFACs generally had disadvantages such as poor anti-interference ability, a high limit of detection, and strong peak tailing. Thus, the quantification of trace PFCAs (i.e., in ≤ppb) in environmental samples is largely reliant on highly sensitive liquid chromatography-mass spectrometry (LC-MS) [7, 10]. Even the samples collected from laboratory batch-processing experiments such as catalytic degradation [11] and toxicological studies [12] are also analyzed with LC-MS. In fact, these samples, generally containing PFCAs in high concentrations (such as in ppm), obtained from laboratory simulation tests are not necessary to rely on expensive LC-MS. In addition to high detection cost, using LC-MS to measure these samples with high-concentration PFCAs is likely to cause residual contamination of the LC-MS pipeline and ion source, increasing the workload of instrumental cleaning for subsequent assays with trace detection requirement. If these laboratory-based samples are diluted in a large proportion before injection, this dilution procedure would be an additive step to introduce possible errors for detection results. Therefore, it is necessary to develop a facile and inexpensive measurement technique suitable for high concentrations of PFCAs from laboratory simulation experiments, e.g., catalytic degradation tests, which are usually massive in sample size.

Up to now, several techniques have been proposed for measuring the samples with high concentrations of PFCAs, which mainly included the following: (1) to introduce UV absorbing or fluorescence groups into the structure of PFCAs and then detect with LC-UV [13] or fluorescence detectors [14] and (2) to increase PFCA volatility *via* derivatization and then detect with gas chromatography (GC) [15]. In contrast, the derivatization followed by HPLC detection is a promising approach since LC is now a widely equipped and easily operated instrument in most laboratories and the carboxylic groups of PFCAs can be easily coupled to some UV-responsive groups through chemical derivatization [16]. To date, several researchers have demonstrated the successful use of precolumn derivatization for LC determination of PFCAs [6, 7]. For example, Qiu et al. used p-bromophenacyl bromide as a derivatization reagent to convert PFCAs into their corresponding esters, and this method could be successfully applied for quantification of PFCAs in environmental samples by combining preconcentration steps [13].  Table 1 lists the LC- UV determination reports of PFACs involving derivatization in recent years. From Table 1, it is clearly seen that the previously used derivatization procedures had limitations such as harsh reaction conditions. We had tried using bromohydroxyacetophenones as derivatization reagents; however, it was found that the derivatization yield of acetophenone reagents was low at room temperature, and the ester derivative was unstable and easily decomposed [16].

Another simple derivatization procedure was purposed for PFCA analysis by GC-MS [19], which used difluoroaniline for derivatization. Stemming from that work, mild derivatization of PFOA followed by LC-UV analysis using inexpensive and easily obtained dichloroaniline as the derivatization reagent was developed by our previous work [16]. By contrast, the amide derivatives would be stable relative to the ester ones. Nevertheless, our reported method was limited to only PFOA analysis, and its derivatization procedure was very inconvenient for requiring post-derivatization cleanup with thin-layer chromatography (TLC). In this work, our goal was to extend the derivatization LC determination to PFCA measurement and additionally, to create more convenient cleanup steps by replacing the TLC with widely applicable solid phase extraction (SPE). The application of the improved derivatization LC method was then assessed by monitoring photocatalytic decomposition of selected perfluorinated environmental pollutants.

## 2. Materials and Methods

### 2.1. Chemicals and Standards

PFCAs (>98% purity) were purchased from Sigma-Aldrich Inc. and included perfluorobutanoic acid (PFBA), perfluoropentanoic acid (PFPeA), perfluorohexanoic acid (PFHxA), perfluoroheptanoic acid (PFHpA), perfluorooctanoic acid (PFOA), and perfluorononanoic acid (PFNA). A precursor of PFOA, perfluorooctane sulfonamide (PFOSA, 98%) was also obtained from Sigma-Aldrich Inc. Dicyclohexylcarbodiimide (DCC) and 3, 4-dichloroaniline (DCA) were purchased from J&K Scientific Ltd (Beijing, China). SPE silica-gel columns (Cleanert Pesticarb-SPE cartridge) were provided by Bonna-Agela Technologies Inc (Tianjin, China). Ultrapure water (18.2 MΩ/cm, Hitech, China) was used for aqueous sample preparation. Possible contamination was avoided by verifying all solids and solvents with procedural blanks in advance.

### 2.2. Derivatization of PFCAs and Post-Derivatization Purification

**Figure 1:**
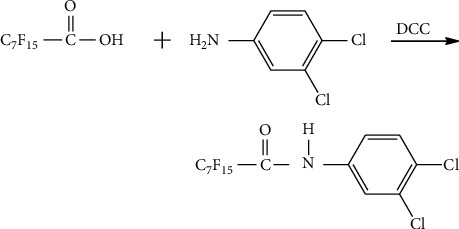
Derivatization reaction of PFOA with DCA by the DCC method.

PFCA derivatization was performed using the classical carbodiimide method [16] to form a series of PFCA-DCA derivatives (Figure 1). Considering that the samples might be dissolved in an organic or aqueous solution, the derivatization reactions in two media were carried out separately.

The method for PFCA-DCA derivative synthesis in an organic medium was as follows: An aliquot of concentrated HCl (5 *µ*L) was added into 1.0 mL tetrahydrofuran solution containing 0.1 mM of a specific PFCA and followed by the addition of 0.1 mL DCC and 0.1 mL DCA (100 mM, both dissolved in tetrahydrofuran). The mixture was stirred at 300 rpm for 1 h at room temperature. The reaction solution was evaporated to dryness using nitrogen. Residues were dissolved in 1.0 mL of a petroleum ether and ethyl acetate solvent system (2 : 0.3, V : V). The further cleanup was performed with an SPE silica-gel column using 20.0 mL of a mixture of petroleum ether and ethyl acetate (2 : 0.3, V : V) as the eluent. The eluent was evaporated to dryness and redissolved into 1.0 mL methanol and measured by LC-UV.

The PFCA-DCA derivatization in an aqueous medium was performed slightly differently from that of the organic phase derivatization. Briefly, 20 mg NaCl and 0.4 mL ethyl acetate were added into 1 mL aqueous solutions of a specific PFCA (0.1 mM). After adding 20 *µ*L of concentrated HCl (to adjust the pH to ∼1.0), 200 *µ*L DCA and 200 *µ*L DCC (100 mM, both dissolved in ethyl acetate) were added to the mixture and stirred at 300 rpm for 1 h, at room temperature, and the organic layer was collected. The residual water phase was re-extracted with ethyl acetate three times and combined with the other organic phases. The combined ethyl acetate extract was evaporated by nitrogen and the subsequent cleanup of SPE was the same as that for the organic medium derivatization as described above.

### 2.3. Separation and Identification of PFCA-DCA Derivatives

Samples containing all PFCA-DCA derivatives were dissolved in methanol and measured on an Agilent 1200-HPLC equipped with a UV detector at the wavelength of 255 nm and a 4.6 × 250 mm SUPELCOSIL LC-18 column (Sigma, USA). The mobile phase was a mixture of methanol and water (86 : 14, V/V) with 1.0 mL/min flow velocity. The identification of PFCA-DCA derivatives was conducted on an Alliance 2695 HPLC equipped with a diode array detector (DAD) and Q-TOF MS (Waters Micromass, UK). The DAD detection wavelength was over a range of 200 to 600 nm. Electrospray ion source in the negative ion ionization mode was used with a scanning range from m/z 150 to m/z 620, the capillary voltage was set to 2.5 kV, the sample cone voltage was 25 V, and the ion source temperature was 120°C. Other parameters of LC-MS were clarified and are shown in our previous study [16].

### 2.4. Methodological Evaluation and Application

Standard curves of PFCA mixtures (0.01, 0.02, 0.05, 0.1, and 0.12 mM of each PFCAs) were carried out with DCA derivatization in organic and aqueous media according to the above-described method, respectively. The instrumental limit of detection of each PFCA was defined as the concentration with a signal-to-noise ratio of 10. Recovery experiments were performed in six parallel experiments with spiked pure water samples (0.01 and 0.12 mM).

Identification and quantification of PFOA and its homologues short-chain PFCAs were made in photocatalytic degradation solutions for photocatalytic degradation tests of PFOA or its precursor PFOSA. The catalytic reactions were conducted according to previously published works [20–22]. Every experiment was conducted with at least three replicates, and each corresponding 1 mL suspension at different reaction points was removed and filtered through a 0.45 *µ*m nylon membrane and derivatized according to the previously described method. All samples were stored at 4°C in the dark until further analysis.

## 3. Results and Discussion

### 3.1. Derivatization Optimization

In preliminary experiments, derivatization was conducted first using bromoacetophenone as the reagent according to the literature [18]. However, the derived product was unstable, and the yield of derivatives was relatively low (less than 30%). As a cheap intermediate derived from the synthesis of pesticides, dyes and drugs, 3,4-dichloroaniline (DCA) can interact with carboxyl groups to form stable amide bonded derivatives [23]. Moreover, DCA derivatization can be conducted in an aqueous phase at mild reaction conditions with the formation of stable derivative products at high yield [24, 25]. Scott et al. developed a precolumn derivatization method using the analog difluoroaniline as a derivatization reagent for PFCA measurement *via* GC-MS [19]. Our preliminary test also indicated that the DCC reaction of PFOA with DCA displayed a high field (>97%), ensuring its satisfactory applicability for PFOA analysis [16]. Herein, DCA, which exhibits strong UV adsorption at ∼255 nm, was chosen as the derivatization reagent in this study.

**Figure 2:**
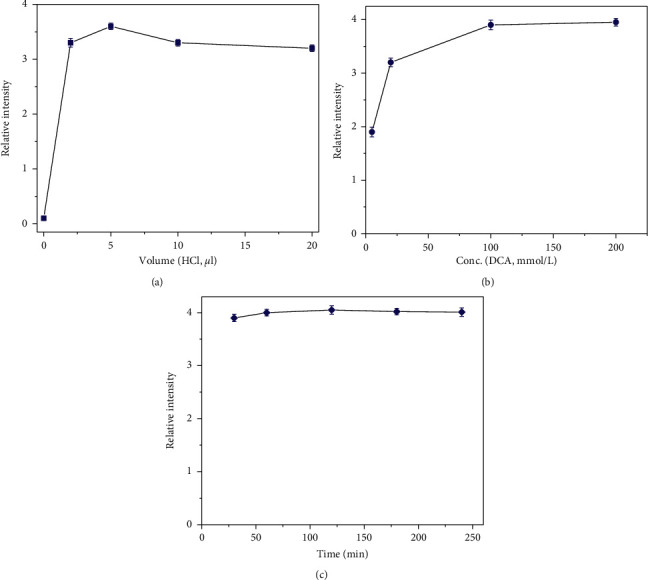
Derivatization optimization: effects of (a) HCl volume, (b) DCA concentration, and (c) reaction time on derivatization yield.

When optimizing the derivative conditions for the organic reaction medium, we found that the choice of a solvent had a strong impact on the yield. Although Hoffman and Liao reported that fatty acids were derivatized in trichloromethane [26], trichloromethane was not suitable for the derivatization of PFCAs due to the low yield. By contrast, ethyl acetate or tetrahydrofuran successfully improved the derivatization yield with derivatization rates of 85% and 97%, respectively. Moreover, solution acidity strongly impacts the derivatization reaction. Ozawa and Tsukioka had reported that the addition of HCl effectively improved the yield in the derivatization of monofluoroacetate with DCC [24]. In this study, when derivatization was carried out with high concentrations (at mg/mL level) of PFCAs in tetrahydrofuran to prepare the derivatives at milligram scales, it was found that there was no need to add HCl. However, when the PFCAs were at a level of mg/L, adding acid into the reaction solution was critical.  Figure 2(a) shows the effect of the added HCl volume on the PFOA-DCA reaction field at a level of 1 mM. With the addition of a volume of concentrated HCl, the yield increased sharply until 5 *µ*L HCl and then decreased slightly with the further addition. Therefore, 5 *µ*L HCl was selected as the optimal dosage, and the concentration and reaction time of DCA were determined under this condition.  Figure 2(b) shows the effect of DCA concentration on the yield of the derivatization reaction. When DCA concentration increased from 5 to 100 mM, the yield increased and with the further increase of DCA concentration, the yield did not continue to increase; so, 100 mM of DCA served as the optimum concentration. However, when the reaction time was between 0.5 and 4 h, the yield only slightly increased (Figure 2(c)). Considering the time required for the entire reaction analysis, 1 h was selected. The derivatization of PFCAs in aqueous media was carried out according to Scott's method [19] with a slight modification as described above.

### 3.2. Establishing the LC-UV Method for Identification and Quantification

**Figure 3:**
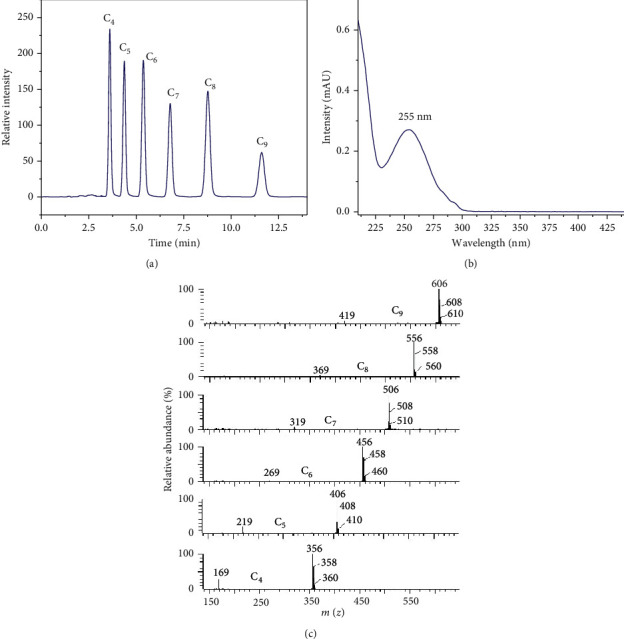
(a) LC chromatogram, (b) UV spectrum, and (c) mass spectra of PFCA-DCA derivatives.

The six PFCA-DCA derivatives (each, 0.1 mM) were dissolved into methanol and separated by HPLC.  Figure 3(a) reveals that HPLC could effectively separate PFCA derivatives and the peak corresponding to PFCAs appeared at the retention time of 2.5–11.7 min. The UV-Vis spectra of PFOA-DCA derivatives (Figure 3(b)) indicated that the maximum absorption peak of the PFCA-DCA derivative was at 255 nm. The mass spectrum of the PFCA-DCA derivative is shown in Figure 3(c), which was consistent with our expectation for the derivatives with the chemical formula (C_*n*_F_2*n*+1_ONHC_6_H_3_Cl_2_) displaying the molecular ion peaks at m/z 606, 556, 506, 456, 406, and 356, all containing two isotope clusters of chlorine, and the corresponding fragments at m/z 419, 369, 319, 269, 219, and 169, respectively, which corresponded to the characteristic ion fragments of [C_8_F_17_]^−^, [C_7_F_15_]^−^, [C_6_F_13_]^−^, [C_5_F_11_]^−^, [C_4_F_9_]^−^, and [C_3_F_7_]^−^. This MS information indicated that the derivatization with DCA was successful.

### 3.3. Quality Control Measures

The relevant parameters of the standard curves are shown in Table S1A. While the linear coefficients (*R*^2^) of standard curves of PFCA-DCA derivatives in the organic medium were all >0.99, the instrumental limits of detection (ILDs) for PFCAs in organic and aqueous mediums were 0.2–0.5 and 0.6–1.5 mg/L, respectively. By contrast, the detection limit of the aqueous phase method was higher because of the lower reaction yield in aqueous phase and the loss of the extraction relative to the derivatization process in the organic phase. Water samples spiked with 0.01 and 0.12 mM PFCAs were used for further methodological assessment. The recoveries and repeatability of DCA derivatization (Table S1B) showed that the recoveries were relatively stable in a range of 66.8%–75.8% in the aqueous phase (0.8% < RSD < 6.8%), 79.9% to 84.6% in the organic phase with RSD in the range of 0.1%–4.2%.

### 3.4. Application in the Analysis of Catalytic Degradation Solutions

**Figure 4:**
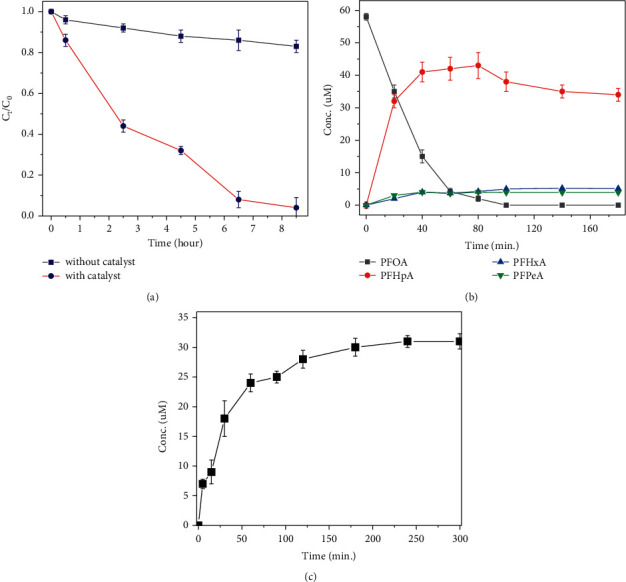
(a) PFOA photodegradation kinetic curves with and without TiO_2_-MWCNT and (b) degradation and product curves of PFOA in the presence of BiOI_0.8_F_2_ at pH 6.0 and 25°C. (c) Degradation and product curves of transformed PFOA from PFOSA in the presence of 0.2 M NaOH and 50 mM potassium persulfate.

At a duration cost of less than 2 h of derivatization followed by purification processing using the inexpensive silica-gel SPE cartridges, the dependence of PFCAs monitoring *via* expensive LC-MS can be removed. This new method was then assessed for its application in the determination of PFCAs in catalytic degradation solutions. In this study, three batch-processing experiments of catalytic degradation of PFOA or its precursor PFOSA were conducted according to the literature [20–22], and the degradation processes were monitored by the derivatization in an aqueous medium followed by LC-UV.  Figure 4(a) shows the PFOA photodegradation kinetic curves with and without a composite TiO_2_ with multiple wall carbon nanotubes (TiO_2_-MWCNT) [20].  Figure 4(b) shows the PFOA degradation and its product intermediates in the presence of BiOI_0.8_F_2_ at pH 6.0 and 25°C [21].  Figure 4(c) displays the PFOA formation curve of its precursor PFOSA in the presence of 0.2 M NaOH and 50 mM persulfate [22]. From Figure 4, it was clearly observed that all of these detection results conferred a similar monitoring effect as using LC-MS, as reported in the literature [20–22].

A suitable preconcentration technology could significantly decrease the method limit of detection by three orders of magnitude [7]. In this study, we had tested a C_18_-SPE procedure to preconcentrate by 1000-fold the spiked water samples before derivatization, it was found that the lowest concentration of PFCAs in water sample can reach 5 *µ*g/L, which was similar to the reported method detection limits in the literature determined by the same LC-UV [13]. However, this derivatization LC-UV method is not recommended to be used for determining the trace amounts of PFCAs in environmental samples, considering that LC-MS is more sensitive and advantageous to exclude false-positive results. On the contrary, this new method was recommended for measuring the samples containing PFCAs in high concentration collected from laboratory batch-processing experiments such as catalytic degradation and toxicology investigation [6, 7].

## 4. Conclusions

In this study, six PFCAs (C_4_–C_9_) were derivatized by a simple, reliable, and effective carbodiimide method before separation and detection by LC-UV. This derivatization procedure possesses the advantages such as mild reaction conditions, facile operation, stable derivatives, enhanced stability, and low cost. We demonstrated that this precolumn derivatization coupled with LC-UV detection has a satisfactory instrumental detection limit, recovery, and repeatability. Finally, we applied this method to successfully monitor PFCAs changing in catalytic degradation solutions, demonstrating that the new method, as a simple and low-cost alternative to LC-MS, can be used for detection of the samples containing PFCAs in high concentration.

## Figures and Tables

**Table 1:** Comparison of HPLC determination of PFCAs coupled with the derivatization procedure.

Targeted species	Derivatization reagents	Solvent medium	Reaction conditions	Detector	Instrumental limit of detection (mg/L)	Reference
PFOA	Benzylamine	Xylene	140°C	UV	20	[17]
PFOA	*α*-Bromoacetophenone	Acetonitrile	80°C	UV	7.0	[18]
PFOA	3, 4-dichloroaniline	Water	Room temperature	UV	0.5	[16]
PFCAs (C_7–11_)	p-Bromophenacyl bromide	Acetonitrile	80°C	UV	5.0	[13]
PFCAs (C_4–9_)	3, 4-dichloroaniline	Water	Room temperature	UV	0.2–0.5	This study

## Data Availability

The “Analytical performances and recoveries of PFCAs via derivatization” data used to support the findings of this study are included within the supplementary information file.

## Supplementary Materials

Supplementary Materials Table S1: analytical performances (A) and recoveries (B) of PFCAs *via* derivatization in organic and water medium.
