# Supplementary material for: Precolumn Derivatization High-Performance Liquid Chromatography for Determination of Perfluorocarboxylic Acids in Catalytic Degradation Solutions
Source: Int J Anal Chem. 2022 May 19;2022:3482759. doi: 10.1155/2022/3482759 (PMC9135559; doi:10.1155/2022/3482759)
Supplement: Supplementary Materials — Table S1: analytical performances (A) and recoveries (B) of PFCAs via derivatization in organic and water medium. [file 3482759.f1.docx]

Supporting information for

Pre-column derivatization high-performance liquid chromatography for determination of perfluorocarboxylic acids in catalytic degradation solutions

Liping Yang ^#^, Binbin Sun ^#^, Haochen Cui, Lingyan Zhu, Guoqiang Shan^^[[1]](#footnote-1)^*^

Key Laboratory of Pollution Processes and Environmental Criteria (Ministry of Education), Tianjin Key Laboratory of Environmental Remediation and Pollution Control, College of Environmental Science and Engineering, Nankai University, Tianjin 300350, P. R. China

**Table S1**. Analytical performances (A) and recoveries (B) of PFCAs *via* derivatization in organic and water medium.

A

|  |  | Organic medium | | | Water medium | | |
| --- | --- | --- | --- | --- | --- | --- | --- |
|  | Concentration  (mM) | Linear equation | Instrument limit of detection  (mg/L) | R^2^ | Linear equation | Instrument limit of detection  (mg/L) | R^2^ |
| PFBA | 0-0.12 | y=9460x-1.22 | 0.2 | 0.99 | y =6270x + 32.3 | 0.6 | 0.99 |
| PFPeA | 0-0.12 | y=8890x-1.41 | 0.2 | 0.99 | y = 6140x -5.90 | 0.6 | 0.99 |
| PFHxA | 0-0.12 | y=11480x-1.15 | 0.2 | 0.99 | y = 7860x-0.96 | 0.6 | 0.99 |
| PFHpA | 0-0.12 | y=123000x-1.17 | 0.3 | 0.99 | y =8850x -6.96 | 0.9 | 0.99 |
| PFOA | 0-0.12 | y=11810x-2.62 | 0.5 | 0.99 | y = 8360.5x -8.20 | 1.5 | 0.99 |
| PFNA | 0-0.12 | y=12190x-1.17 | 0.5 | 0.99 | y = 84500x + 10.6 | 1.5 | 0.99 |

B

|  | **Sample (mM)** | **^a^Recovery (%)** | **^b^Recovery (%)** |
| --- | --- | --- | --- |
| PFBA | 0.12 | 74.8±1.1 | 84.6±0.1 |
|  | 0.01 | 72.3±4.2 | 81.3±2.2 |
| PFPeA | 0.12 | 72.9±0.8 | 83.9±0.8 |
|  | 0.01 | 73.9±3.9 | 79.9±1.9 |
| PFHxA | 0.12 | 71.4±1.0 | 82.4±0.2 |
|  | 0.01 | 75.7±3.4 | 81.7±2.4 |
| PFHpA | 0.12 | 73.0±9.2 | 83.0±0.2 |
|  | 0.01 | 71.8±4.8 | 80.8±2.8 |
| PFOA | 0.12 | 70.7±6.3 | 82.7±0.3 |
|  | 0.01 | 69.9±6.8 | 81.9±2.8 |
| PFNA | 0.12 | 68.9±1.7 | 83.9±1.7 |
|  | 0.01 | 69.3±4.2 | 80.3±4.2 |

^a^ derivatizition in aqueous medium; ^b^ derivatizition in organic medium.

1. * Corresponding author, E-mail address: [yunongshan@nankai.edu.cn](mailto:yunongshan@nankai.edu.cn). Phone: +86-22-23500791. Fax: +86-22-23503722. ^#^L.Yang and B. Sun contributed equally to this work. [↑](#footnote-ref-1)
